# Primary papillary epithelial tumor of the sella harboring an *EZH2* Y646F mutation

**DOI:** 10.1007/s00401-025-02910-6

**Published:** 2025-07-15

**Authors:** Jawad Fares, Pouya Jamshidi, Harrshavasan T. Congivaram, Daniel Oyon, Melissa Mejia Bautista, Robert C. Kern, Daniel J. Brat, Mark W. Youngblood, James P. Chandler

**Affiliations:** 1 Department of Neurological Surgery, Feinberg School of Medicine, Northwestern University, Chicago, IL 606011 USA; 2 Northwestern Medicine Malnati Brain Tumor Institute, Robert H. Lurie Comprehensive Cancer Center, Chicago, IL 60611 USA; 3 Department of Pathology, Feinberg School of Medicine, Northwestern University, Chicago, IL 60611 USA; 4 Department of Otolaryngology, Feinberg School of Medicine, Northwestern University, Chicago, IL 60611 USA

Sellar and suprasellar tumors encompass a heterogeneous array of neoplasms, including PitNETs (pituitary neuroendocrine tumors)/adenomas, craniopharyngiomas, meningiomas, and metastatic lesions. Among these, primary papillary epithelial tumor of the sella (PPETS) is a newly proposed and underrecognized entity distinguished by its papillary architecture, immunophenotypic profile, and emerging molecular characteristics that are beginning to be defined in recent studies. Not currently classified within the World Health Organization (WHO) framework, PPETS presents a diagnostic challenge due to its overlap with other sellar and metastatic epithelial neoplasms. Histologically, these tumors typically exhibit papillary architecture of columnar epithelial cells and demonstrate strong nuclear expression of thyroid transcription factor-1 (TTF-1) [1, 11]. 

The enhancer of zeste homolog 2 (*EZH2*) encodes the catalytic subunit of the polycomb repressive complex 2 (PRC2), which epigenetically silences genes through trimethylation of histone H3 at lysine 27 (H3K27me3). Mutations in *EZH2*, most notably the Y646F hotspot substitution in the SET domain, enhance its methyltransferase activity, leading to increased global H3K27me3 levels and repression of tumor suppressor genes [2, 3, 5]. *EZH2 *Y646F has been well characterized in follicular lymphoma, diffuse large B cell lymphoma, and select epithelial malignancies such as endometrial, prostate, and breast carcinomas. However, it has not been described in papillary carcinomas of the thyroid or lung, which represent the main differential diagnoses of PPETS, nor has it been previously reported in tumors of the sellar region.

We present a unique case of a 47-year-old male with a sellar and suprasellar mass that, despite thorough systemic evaluation, lacked an identifiable primary origin. Magnetic resonance imaging (MRI) revealed a relatively large heterogeneously enhancing lesion with increased T1 signal and susceptibility artifact suggestive of hemorrhage (Fig. 1a, b). The mass extended into the suprasellar cistern, displaced the optic chiasm, and involved the cavernous sinuses bilaterally up to the intercarotid line. There was direct contact with the supraclinoid and parasellar segments of the internal carotid arteries, and the mass partially effaced the prepontine cistern. Laboratory evaluation showed elevated prolactin levels (22.6ng/mL), consistent with stalk effect.

**Fig. 1:**
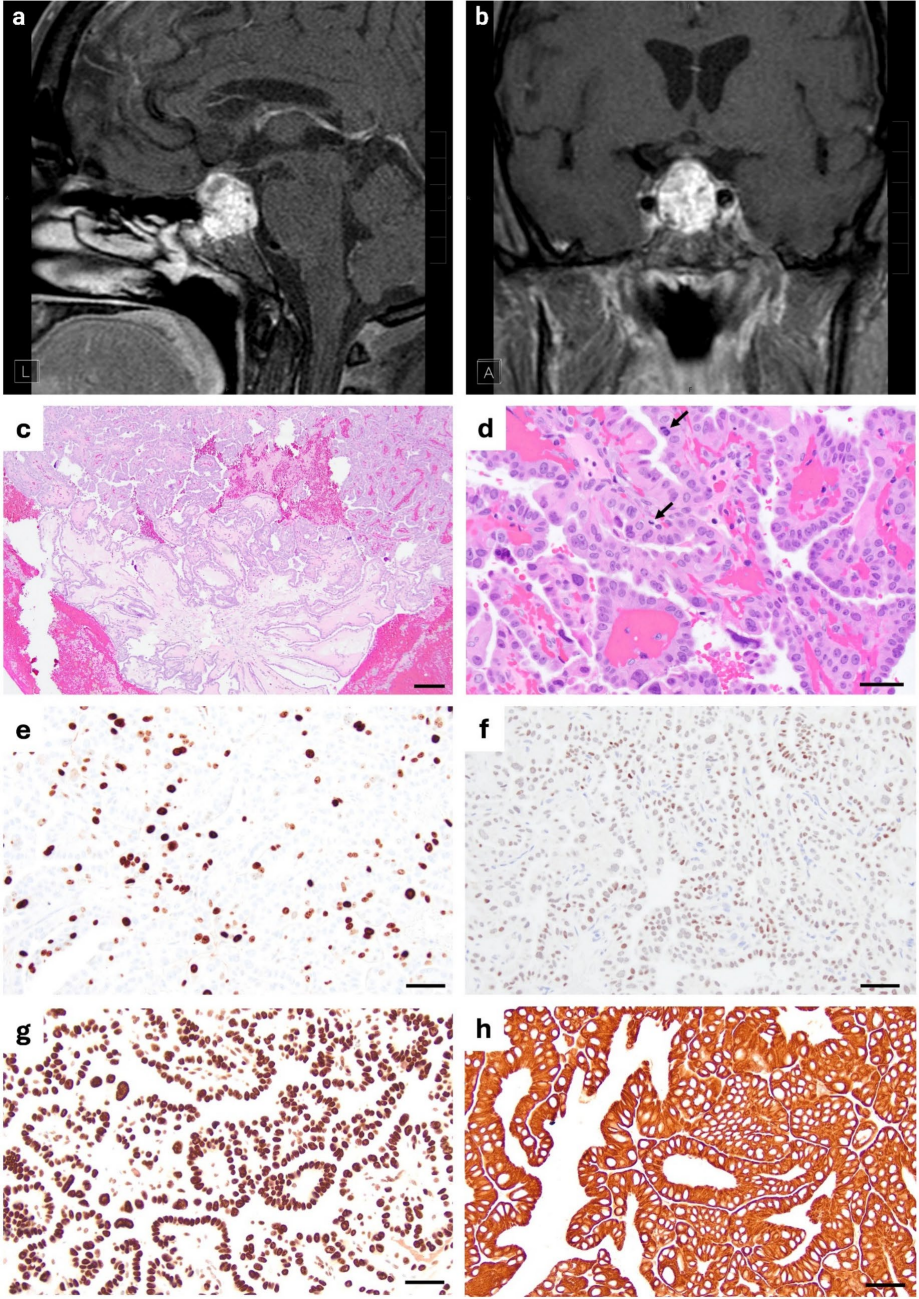
Radiological and histopathological features of a primary papillary epithelial tumor of the sella (PPETS). **a**, **b** Sagittal and coronal T1-weighted post-contrast MRI showing a heterogeneously enhancing sellar/suprasellar mass. **c** H&E-stained section reveals papillary architecture with columnar epithelial cells surrounding fibrovascular cores (bar = 200 µm). **d** Tumor cells exhibit moderate pleomorphism, irregular nuclear contours, and conspicuous mitotic activity (arrow; bar = 20µm). **e** Ki-67 immunostaining highlights focal proliferation up to 15% (bar = 50 µm). **f** Immunohistochemistry demonstrates strong nuclear TTF-1 expression, **g** retained nuclear expression of H3K27me3, and **h** diffuse membranous CK7 labeling (bar = 50 µm)

The patient underwent endoscopic endonasal resection with frameless stereotactic navigation. Intraoperatively, the tumor appeared firm, infiltrative, and inseparable from the pituitary gland and diaphragm sellae. Subtotal resection was achieved using standard microsurgical techniques. Postoperative MRI showed significant tumor debulking with minimal residual enhancement and decompression of the optic chiasm. Given the tumor’s proximity to critical structures and the incomplete resection, the patient underwent Gamma Knife radiosurgery to the residual lesion. Follow-up imaging over several months demonstrated continued contraction of the tumor cavity and resolution of mass effect.

Histopathologic analysis revealed an epithelial neoplasm with papillary architecture composed of single to multilayered columnar cells lining fibrovascular cores (Fig. 1c). The tumor demonstrated nuclear atypia, prominent nucleoli, and mitotic activity measured at 2 mitoses per mm^2^ (Fig. 1d), raising concern for malignant potential. The Ki-67 labeling index was initially estimated semi-quantitatively at approximately 12–15% of tumor cells. Quantitative manual counting yielded a proliferation index of 10.97% (89 positive cells out of 811 tumor cells) (Fig. 1e). Immunohistochemistry was positive for CK7 and TTF-1 and negative for PAX8, Napsin A, synaptophysin, and PD-L1 (Fig. 1f–h). TTF-1 immunostaining was performed using a mouse monoclonal antibody (clone 8G7G3/1, Millipore Sigma; dilution 1:100). Immunohistochemical staining for H3K27me3 showed retained nuclear expression in tumor cells, consistent with the activity of the *EZH2 *Y646F mutation.

Due to the tumor’s ambiguous morphology and immunophenotype, further molecular testing was performed. To elucidate the tumor’s epigenomic profile, DNA methylation profiling was performed using Illumina EPIC arrays and analyzed against four independent classifiers with libraries composed of CNS tumors and tumors of unknown origin (TUO). The NIH/NCI classifier reported high confidence scores for epithelial tumors (0.96) and lung adenocarcinoma (0.90), while the Northwestern tumor of unknown origin (NMH TUO) classifier [4] showed no classification match, but on t-SNE plot, clustered closest to thyroid carcinoma. The tumor received a low methylation score (0.38) to posterior pituitary tumors (pituicytoma, granular cell tumor, spindle cell oncocytoma) on the DKFZ v12.8 classifier. Similarly, the tumor did not match a known methylation class on the Northwestern CNS tumor classifier (NMH CNS) [13], but clustered closest to the same posterior pituitary tumor group (Fig. S1). This result suggests an epigenetic proximity to a tumor arising in the sellar region, posterior pituitary, or infundibulum. This finding correlates well anatomically with our case. The shared TTF-1 immunoreactivity across posterior pituitary neoplasms, thyroid, and lung adenocarcinomas likely influenced epigenetic similarity in classifiers [6, 8]. Conflicting epigenetic results on independent classifiers with TUO methylation library and the lack of a calibrated match score to CNS tumor library highly suggest that this primary tumor is not recognized as a known tumor type. This result is further supported by the lack of evidence for other malignancy on systemic imaging, and the patient’s stable post-operative clinical course.

Copy number variation analysis derived from the methylation array revealed heterozygous segmental loss of chr9q and gain at ch21p (Fig. 2A). Next-generation sequencing using the PGDx Elio Tissue Complete panel identified two pathogenic variants: *EZH2 *Y646F and *BRCA2 *N136fs. The *EZH2* mutation, affecting the SET domain, is a gain-of-function alteration known to enhance PRC2-mediated chromatin silencing [2, 7, 14]. The tumor was microsatellite stable with a tumor mutational burden (TMB) of 9.2 mutations/Mb (Fig. 2B). RNA-based fusion analysis using the FusionPlex panel detected no gene rearrangements.

**Fig. 2:**
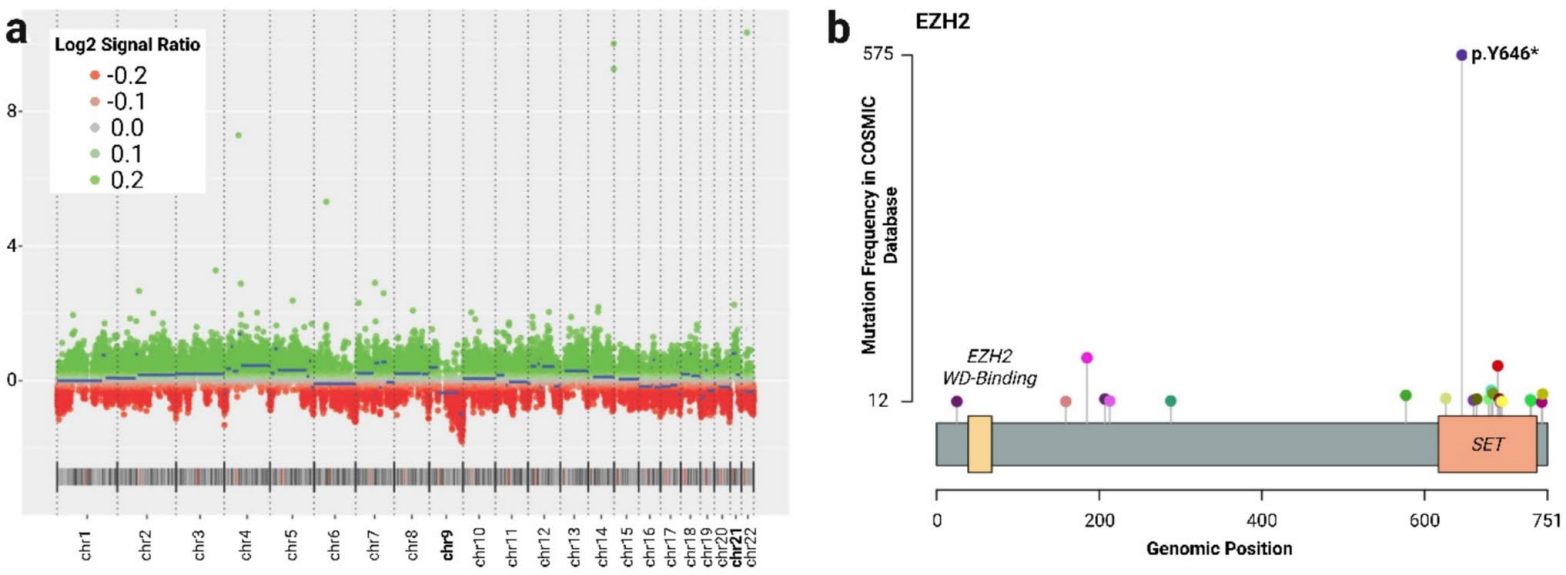
Molecular characteristics of a primary papillary epithelial tumor of the sella (PPETS). **A** DNA methylation-based copy number variation shows heterozygous segmental loss of chr9q and gain of ch21p. **B** Lollipop plot shows the *EZH2* Y646X hotspot mutation within the SET domain, with substitution frequency mapped across the gene (COSMIC database)

In the absence of a detectable primary malignancy and based on integrated radiologic, histologic, and molecular findings, the tumor was classified as a PPETS. This diagnosis is consistent with recent literature describing TTF-1–positive sellar tumors with papillary architecture and no identifiable primary site [1, 11]. The presence of the *EZH2 *Y646F mutation, not previously reported in this context, suggests a novel molecular subtype of PPETS with potential therapeutic relevance. Additionally, the *BRCA2* frameshift mutation (variant allele frequency: 53%) raises concern for an underlying hereditary cancer predisposition syndrome, warranting genetic counseling.

Roncaroli et al. initially proposed PPETS as a distinct tumor entity after identifying TTF-1–positive epithelial tumors of the sella that lacked features of recognized sellar neoplasms [10]. These tumors exhibited uniform cytokeratin and TTF-1 expression but lacked clear lineage markers for lung or thyroid origin. Subsequent reports, including that of Rima et al. [9], supported the classification of PPETS as a novel entity and emphasized their often bland histologic appearance and low proliferative indices. In contrast, our case demonstrates a high proliferative and relatively atypical cytomorphology, suggesting that PPETS likely extends along a broader histopathologic and biological continuum.

Feng et al. [5] expanded on these findings by performing an integrated transcriptomic and epigenomic analysis of PPETS and posterior pituitary tumors. Their data revealed overlapping methylation and gene expression profiles, leading them to propose that these tumors comprise a single neuro-oncological entity. However, their study did not report *EZH2* mutations, underscoring the uniqueness of our case and the potential for molecular stratification within this emerging group. While our case is the first to report an *EZH2 *Y646F mutation in a PPETS, further studies are needed to determine the prevalence and diagnostic relevance of this alteration across a broader cohort of PPETS. Confirmation in additional cases would support its potential role in molecular stratification of this rare tumor entity.

Therapeutically, the *EZH2 *Y646F mutation is of particular interest. This gain-of-function alteration enhances PRC2 activity, silencing key tumor suppressor pathways. Pharmacologic inhibitors of EZH2, including GSK126, EPZ6438, and the FDA-approved agent tazemetostat, have demonstrated efficacy in tumors bearing this mutation [2, 7, 9]. While their role in PPETS remains unexplored, our findings suggest that EZH2-targeted therapies may represent a rational approach in cases of progression or recurrence.

The co-occurrence of a *BRCA2 *N136fs truncating mutation implicates defective homologous recombination, opening the possibility for treatment with poly (ADP-ribose) polymerase (PARP) inhibitors. These agents are now standard in BRCA-mutant breast, ovarian, prostate, and pancreatic cancers and may have activity in CNS tumors with similar defects [12], though this remains to be tested. The absence of PD-L1 expression and modest TMB suggests limited potential for immune checkpoint blockade in this case.

Altogether, this case illustrates the diagnostic uncertainty surrounding tumors not captured by current WHO classifications, while demonstrating the value of integrated histopathological and molecular analysis. It expands the clinicopathologic spectrum of PPETS and, to our knowledge, is the first report of an *EZH2 *Y646F mutation in a sellar tumor. The coexistence of epigenetic and DNA repair alterations raises the possibility of a molecular subset, warranting further study. Comprehensive profiling should be considered in ambiguous sellar tumors, as it may reveal actionable targets and guide therapy.

## Supplementary Information

Below is the link to the electronic supplementary material.

Supplementary file1 (DOCX 289 KB)
