# Supplementary material for: Primary papillary epithelial tumor of the sella harboring an EZH2 Y646F mutation
Source: Acta Neuropathol. 2025 Jul 15;150(1):4. doi: 10.1007/s00401-025-02910-6 (PMC12263792; doi:10.1007/s00401-025-02910-6)
Supplement: Supplementary file 1 — Supplementary file1 (DOCX 289 KB) [file 401_2025_2910_MOESM1_ESM.docx]

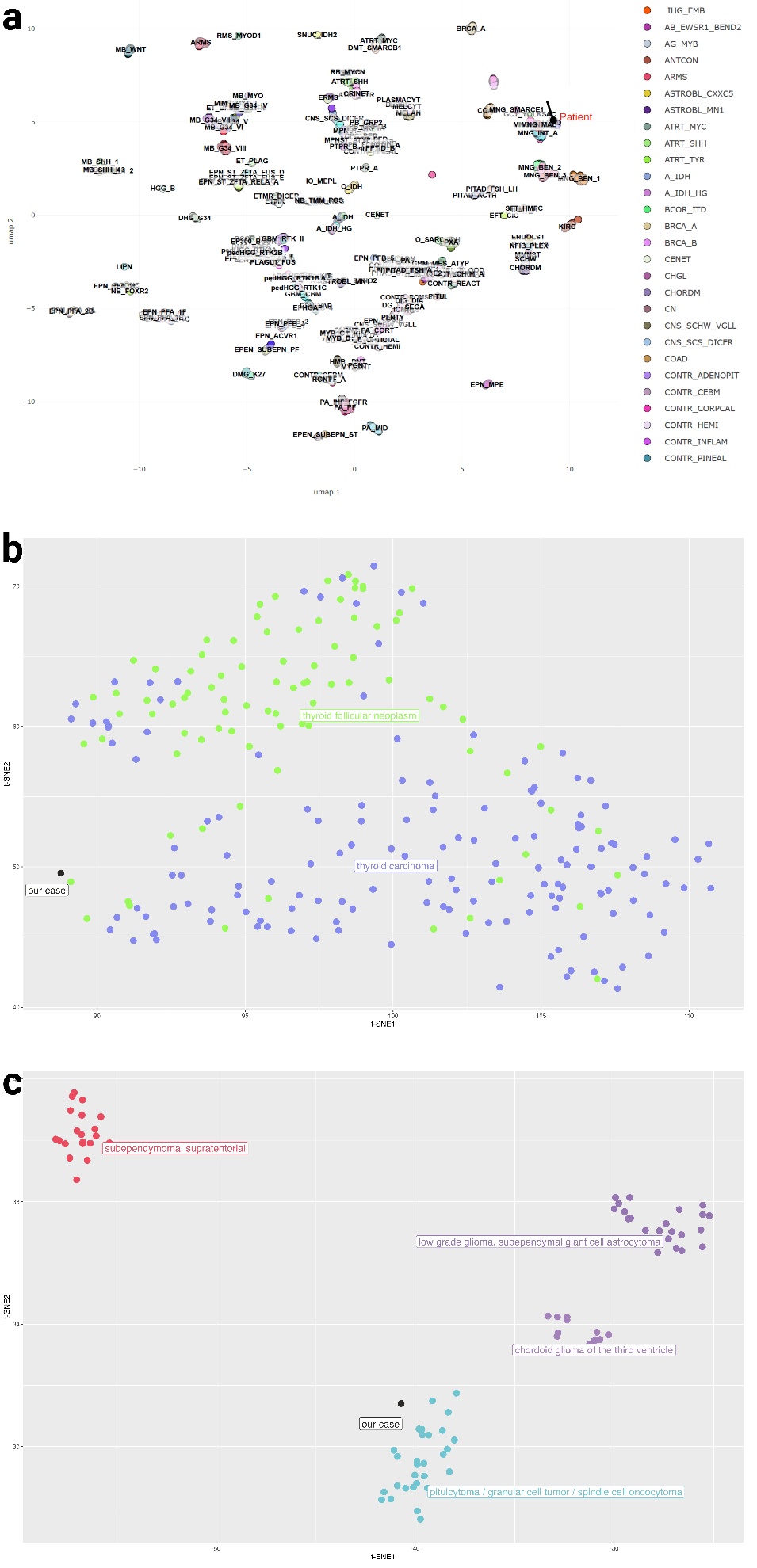


**Figure S1. Epigenomic analysis of the tumor using multiple methylation classifiers and dimensionality reduction approaches. (a)** The NIH/NCI classifier returned high-confidence (though non-calibrated) scores for epithelial tumors (0.96) and lung adenocarcinoma (0.90), positioning the tumor near lung adenocarcinoma on UMAP projection. **(b)** The Northwestern tumor of unknown origin classifier did not yield a match but clustered the tumor closest to thyroid carcinoma on t-SNE. **(c)** The Northwestern CNS tumor classifier did not yield a match but placed the tumor closest to a cluster of posterior pituitary tumors (pituicytoma, granular cell tumor, spindle cell oncocytoma).
